# How to spot the truth

**DOI:** 10.1113/EP092160

**Published:** 2024-08-07

**Authors:** G. Drummond, M. J. Tipton

**Affiliations:** ^1^ Anaesthesia, Critical Care and Pain Medicine University of Edinburgh Edinburgh UK; ^2^ Extreme Environments Laboratory University of Portsmouth Portsmouth UK

## 1 INTRODUCTION

‘Truth’ is under attack, more so now than ever before, and for many reasons one of which is social media. We hear and read remarkable, often preposterous claims from many sources. This may be in political debate, the presentation of new products, or new health‐enhancing exercises ranging from hot water pools to cold water swimming. These frequently claim to be ‘scientific findings’ often reporting ‘new studies have shown’ stories, underpinned by ‘expert’opinion. They are amplified in the media until the next fad comes along.

This pervasive form of persuasion is a war of beliefs, which in many cases may contradict accepted knowledge. It is always possible, in fact likely, that some of the more absurd claims may not involve, or even be properly aware of, current scientific understanding, in which case these claims may be logical, but based on incorrect assumptions or understanding. Flat earthers have a consistent world view, which is probably logical to them; it just is not compatible with other known facts. But truth is the first casualty of war, and now more than ever, we must equip ourselves and others with the skills needed to judge how valid the information we are presented with is.

This is not as simple as it might appear. The context is all‐important. Interestingly, there are far fewer exact rules, firm guidelines and exact cut‐off levels than people might imagine for establishing the truth. Most scientific knowledge is rarely expressed in terms of utter validity, but rather expressed as ‘fits’ or ‘is not inconsistent with’ what we know already, or ‘suitable for predicting performance’. For example, we now know that gravity can be bent; but Newton's simple straight‐line approximation has taken astronauts to the moon and back (sorry, flat earthers). In addition, although statisticians use words consistently and exactly, they do not use words such as ‘population’ and ‘sample’ in the way they are used in general parlance. Nor is the logic of statistics straightforward. For example, the most commonly used tests of likelihood assume ‘if, and only if, these random samples were drawn from a single population, then…’ Logical and consistent, yes, but not well understood, even by some scientists. For example, in one study, trainee doctors, who should be reading this sort of stuff all the time, were given a simple statement using this test. When asked to choose the correct conclusion out of four possibilities, almost half made a wrong choice (Windish et al., 2007).

## 2 WHY IS GETTING AS CLOSE AS POSSIBLE TO THE TRUTH IMPORTANT?

The truth helps you make ‘adequately correct’ decisions and act accordingly. Such decisions depend on the situation, and the risks of making a correct or incorrect decision. Uncertainty doesn't mean we know nothing, or that anything could be true: it just means you don't bet your house on an outsider.

Some years ago, a district court decided that a particular vaccine was responsible for an adverse outcome (which was scientifically doubtful). This triggered a disastrous decrease in child vaccinations for a whole range of diseases. It also showed convincingly that the transmission of the faulty conclusion was related to internet broadband access: more broadband, greater decrease in vaccinations (Carrieri et al., 2019).

In another case, however, a US court rejected a manufacturer's defence that there were insufficient data to meet the usual scientific criteria to demonstrate a causal link between a drug and a serious, but rare, adverse event; and this is why the drug was marketed without a warning. The court was unwilling to accept this statistical threshold, preferring to heed the reports of infrequent, but important, adverse events after the use of the drug, and thus awarded damages (Matrixx initiatives, Inc. et al. vs Siracusano et al., 2011).

Here, we shall try to show the reader the processes applied in scientific evaluation, in the hope that you can apply them in your day‐to‐day decision‐making. Facts don't speak for themselves—context is vital. An experienced scientist, who “knows the ropes”, is more likely to use their knowledge, experience and judgement to tease out the full story. The central question is not ‘can we be certain?’, but rather ‘can we process this information and adjust our ideas?’ Uncertainty is always present, but we may be able to be ‘confidently uncertain’.

## 3 A CHECKLIST FOR TRUTH (ELEMENTS OF THE CONTEXT AND QUESTIONS THAT SHOULD BE ASKED OF ANY CLAIM)

- Who is making the statement, and what is their qualification for making it?
- What was the original question? Has it been correctly framed?
- What is the underpinning evidence for the statement? What is the provenance of the supporting data? Where has it been published? Are there alternative explanations, have these been explored, how possible are they?
- Has the best measure been used? The best way to express ‘typical’ is as the median value, as is done by the Office for National Statistics. However, many reports use the average, which could be far from the same thing and make, for example, the ‘typical’ person apparently better off (if we put incomes in order of size, from the least to the greatest, the ‘median’ is the one closest to the halfway point in this order. Many more incomes are small, only a few are whopping, so the median is closer to the bottom. The ‘average’ or ‘mean’ is the sum of *all the money* in the incomes [lots of paltry ones, some whopping ones] divided by all the incomes considered in the sample. For example, median UK household disposable income in the financial year ending 2022 was about £32K, and the average was £40K.)
- Have basic scientific principles been used: for example, how was the sample of people that was tested obtained? The concept of a ‘random’ sample, scientifically, is that it will contain people from all walks of life, ages, states of health of the target population: so that the results can be applied to that population. If we study healthy students, then the answer may only apply to healthy students.
- Were sufficient people tested to reliably and confidently find an effect? The most reliable and frequent (but rather clumsy) study design is a ‘randomised controlled trial', often used to test new drugs against old ones. Such studies often need hundreds of participants if the drugs aren't that different in effect. Smaller studies may not reliably find an effect: if they do, by chance, then this change exaggerates the benefit (this is known as the ‘winner's curse’ [Sidebotham & Barlow, 2024]—attempts to verify or replicate this first observed effect often fail!).
  It is not easy to prove that something does not exist, and a large study is needed to reach valid conclusions. This is important if you are investigating a rare but serious complication or a new technique. For example, if a new surgical procedure is carried out 20 times without a problem, it is not necessarily safe. If the same procedure were carried out 100 times, *and the death risk were randomly distributed in the same way as for the first 20*, there is a 95% chance that the number of deaths will be between 0 and 16 (and it is likely that fitter patients were selected first in the original study—see ‘ bias’ below).
- Was there a ‘control group’? If an intervention is being assessed (e.g., the health benefits of cold‐water swimming), then a control group is needed that will carry out the same activities but without the hypothesised ‘active ingredient’ (e.g., cold). The control group should include all other factors that could be at work, such as similar locations, similar companions, same food, same exercise, same bedtime and sleep profile, etc.
- Humans vary a great deal, so experiments comparing human participants are difficult. This is particularly obvious in responses to medication, and can lead to unexpectedly different results. An elegant way of getting around this is to ‘cross‐over’ a treatment and compare the same individuals, each given both the ‘control’ and the ‘active’ treatment. However, without care this can also lead to complexities. Ideally half the participants should start with the active treatment, and half with a ‘neutral’ (control) treatment, but how can we be sure that the active treatment has worn off (‘washed out’) before testing the control treatment? For example, hormones may have effects that last long after the actual drug has left the body, and some psychophysiological changes can be long‐lasting. Indeed, some would argue that, in some studies, with some people, wash out may never fully occur (Tipton & Mekjavic, 2000).
- What measurements are made? Are these measurements, like blood pressure, blood levels of hormones? Or questionnaires? What questions get asked? It is very easy to ask leading questions, particularly if the person taking part believes something is doing them good. A far better (but far less likely) outcome would be health assessments a year after an intervention! Do the scientists making the measurements know the treatment, and what do they expect to find? In one study, when a pain‐killer was tested, the testers (who were kept unaware of the drug being tested) found different effects if the tester had different expectations of the drug's effects (Gracely et al., 1985).
- Are tests being used as ‘proxy’ or ‘surrogate’ measurements for something that is more important but not as easy to measure? Examples include using exam scores as an index of ability, or body mass index (BMI) for health assessments. How reliable, and exact, are such surrogate assessments?
- Does the proponent have any conflict of interest? Does what they argue benefit them?
- Is there any ‘bias’? Bias can creep in at lots of stages in the process of getting information and presenting it. Scientific publications are very varied: papers in highly regarded journals have met demanding acceptance standards, with stringent peer assessment, compared with some ‘open access’ journals, where papers are also assessed, but the author pays, or ‘vanity journals’ where the author only has to pay to get published! However, all journals are looking to attract readers and citations, and there is nothing better than controversy to boost readership and citations. Additionally, presentations at conferences often turn up as ‘publications’ but have had virtually no peer assessment, and such conferences can be international, national or local.
- The funding of research affects what gets published. Published research papers funded by companies and dealing with available products are more likely to give a “positive” result than studies independently funded (Bourgeois et al., 2010). Product evaluation can be designed to be flattering in terms of the variables assessed, avoiding observing later adverse effects, and selecting those tested (age, sex, race). It is now necessary to register clinical studies before they start: but lots of studies funded by drug companies are not published. Even trivial effects can be ‘statistically significant’ if the study is large enough. Regulatory oversight of large scale, urgent studies can be limited and poor practice can be concealed (Powell‐Smith & Goldacre, 2016).
- Survival bias is relevant. Are the data already selected? A salutary application of the study of survivors was the analysis of damage found on aircraft returning to base after combat. Clearly, a returning aircraft could take damage in those areas and still fly well enough to return safely to base. Thus, it would be best, if possible, to protect areas that were not seen to be damaged in these aircraft. Hits in undamaged areas presumably were more crippling (Mangel & Samaniego, 1984).

Overall, as a result of failure to meet some of the requirements listed above, about half of published medical papers are unlikely to be true (Ioannidis, 2005). In 2023, the number of retractions for research articles internationally reached a new record of over 10,000 (Noorden, 2023) due to an increase in sham papers and peer‐review fraud. Furthermore, despite a requirement for disclosure, a lot of government research is never released, or is delayed until interest in the topic has declined.

A recent study (Briganti et al., 2023) reviewed the papers published on the health and recovery benefits of cold‐water exposure. They found 931 articles, and then carefully weeded out irrelevant studies. The authors were left with 24 papers, and in these the risk of bias was ‘high’ in 15 and ‘gave concern’ in four. Thus, only five papers had a ‘low’ risk of bias: three of these looked at cold water immersion after exercise and two at cognitive function. So, a very small percentage of the studies examined had anything really useful to say.

## 4 WHAT ABOUT THE ‘FINDINGS’ YOU ARE PRESENTED WITH?

Watch out for percentages (Bolton, 2023). A simple change is easily understood as a percentage, but ‘scientific’ studies involving comparisons between groups can require more careful consideration. These comparisons should always trigger the question ‘percentage of what, exactly?’ The headline, ‘New drug/product/intervention cuts mortality by 50%’ sounds impressive, and attracts attention, but the reality could be less spectacular. Perhaps using the old drug, the death rate was 20 per 1000 patients, and when the new drug was first used, the rate became 10 per 1000 patients: a 50% reduction. But the absolute risk reduction in death rate was 10 per 1000, or 1%, a less impressive headline.

Also, beware of correlations. Just because two things relate to each other, for example, a diet and a sense of well‐being, does not mean that one causes the other. The world is full of accidental (spurious) correlations (Van Cauwenberge, 2016). One of our favourites is the high correlation between the divorce rate in Maine, USA and the per capita consumption of margarine! Also, ask the question ‘how many false positives and negatives will I get if I use this correlation to make a decision’ (Tipton et al., 2012).

For the moment at least, artificial intelligence cannot quantify uncertainty very well. Generally, AI uses stuff from ‘out there’ as if it were true. Thus, a high proportion of garbage in will give you garbage out (which increases the proportion of garbage that AI uses next time round)!

We hope that, armed with the above checklist, you can challenge and interrogate the polarising information, from ‘spin’ to the outright falsehoods presented to you on a daily basis. We are at risk of being overwhelmed by an increasing number of dubious, unregulated and disparate sources. The next time you hear phrases like ‘they say this is great’ or ‘this is scientifically proven’ start by asking ‘who are they?’ and ‘which scientists, using which methods?’ Be cautious and questioning; snake oil and its vendors still exist, they come in many guises.

## AUTHOR CONTRIBUTIONS

M. J. Tipton conceived the work. Both authors contributed to the design of the work, acquisition, analysis, or interpretation of data for the work, drafting of the work or revising it critically for important intellectual content. They both approved the final version of the manuscript and agree to be accountable for all aspects of the work in ensuring that questions related to the accuracy or integrity of any part of the work are appropriately investigated and resolved. All persons designated as authors therefore qualify for authorship, and all those who qualify for authorship are listed.

## CONFLICT OF INTEREST

None declared.

## FUNDING INFORMATION

No funding was received for this work.

## References

[eph13614-bib-0001] Bolton, P. (2023). House of Commons Library Research Briefing. How to spot spin and inappropriate use of statistics. https://commonslibrary.parliament.uk/research-briefings/sn04446/

[eph13614-bib-0016] Bourgeois, F. T. , Murthy, S. , & Mandl, K. D. (2010). Outcome reporting among drug trials registered in ClinicalTrials.gov. Annals of Internal Medicine, 153(3), 158–166. 20679560 10.1059/0003-4819-153-3-201008030-00006 PMC3374868

[eph13614-bib-0002] Briganti, G. L. , Chesini, G. , Tarditi, D. , Serli, D. , & Capodici, A. (2023). Effects of cold water exposures on stress, cardiovascular, and psychological variables. Acta Physiologica, 239(4), 1–4. 10.1111/apha.14056 37840386

[eph13614-bib-0003] Carrieri, V. , Madio, L. , & Principe, F. (2019). Vaccine hesitancy and (fake) news: Quasi‐experimental evidence from Italy. Health Economics, 28(11), 1377–1382. 31429153 10.1002/hec.3937 PMC6851894

[eph13614-bib-0004] Gracely, R. H. , Dubner, R. , Deeter, W. R. , & Wolskee, P. J. (1985). Clinicians' expectations influence placebo analgesia. The Lancet, 325(8419), 43. 10.1016/s0140-6736(85)90984-5 2856960

[eph13614-bib-0005] Ioannidis, J. P. A. (2005). Why most published research findings are false. PLoS Medicine, 2(8), e124. 16060722 10.1371/journal.pmed.0020124 PMC1182327

[eph13614-bib-0006] Mangel, M. , & Samaniego, F. (1984). Abraham Wald's work on aircraft survivability. Journal of the American Statistical Association, 79(386), 259–267. Reprint on author's web site Archived 2019‐08‐17 at the Wayback Machine.

[eph13614-bib-0007] Matrixx initiatives, Inc., et al. vs Siracusano et al . (2011). United States court of appeals for the ninth circuit No. 09–1156. Decided March 22. https://supreme.justia.com/cases/federal/us/563/27/

[eph13614-bib-0008] Noorden, R. V. (2023). More than 10,000 research papers were retracted in 2023 — A new record. Nature, 624(7992), 479–481. 38087103 10.1038/d41586-023-03974-8

[eph13614-bib-0009] Powell‐Smith, A. , & Goldacre, B. (2016). The TrialsTracker: Automated ongoing monitoring of failure to share clinical trial results by all major companies and research institutions [version 1; peer review: 2 approved]. F1000Research, 5, 2629. 28105310 10.12688/f1000research.10010.1 PMC5200941

[eph13614-bib-0010] Sidebotham, D. , & Barlow, C. J. (2024). The winner's curse: Why large effect sizes in discovery trials always get smaller and often disappear completely. Anaesthesia, 79(1), 86–90. 37889911 10.1111/anae.16161

[eph13614-bib-0011] Tipton, M. J. , & Mekjavic, I. B. , & Eglin, C. M. (2000). Permanence of the habituation of the initial responses to cold‐water immersion. European Journal of Applied Physiology, 83(1), 17–21. 11072768 10.1007/s004210000255

[eph13614-bib-0012] Tipton, M. J. , Milligan, G. S. , & Reilly, T. J. (2012). Physiological employment standards1. Occupational fitness standards: Objectively subjective? European Journal of Applied Physiology, 113(10), 2435–2446. 23263741 10.1007/s00421-012-2569-4

[eph13614-bib-0013] Van Cauwenberge, L. (2016). https://www.datasciencecentral.com/spurious‐correlations‐15‐examples/

[eph13614-bib-0014] Windish, D. M. , Huot, S. J. , & Green, M. L. (2007). Medicine residents' understanding of the biostatistics and results in the medical literature. Journal of the American Medical Association, 298(9), 1010–1022. 17785646 10.1001/jama.298.9.1010

